# What Is Your Diagnosis? Large Mediastinal Mass in a Dog

**DOI:** 10.1111/vcp.70064

**Published:** 2025-10-15

**Authors:** Rosane O. Cruz, Talles M. Almeida, Iris D. S. Menezes, Ludmila R. Moroz, Suane N. Boaventura, Tiago Peixoto, Stella F. Valle, Nicole Hlavac

**Affiliations:** ^1^ Department of Anatomic Pathology and Veterinary Clinics, Veterinary Medicine Hospital Universidade Federal da Bahia Salvador Brazil; ^2^ Department of Veterinary Clinical Pathology Universidade Federal do Rio Grande do Sul Porto Alegre Brazil

**Keywords:** Alcian Blue stain, extraskeletal myxoid tumor, mucin

## 1 Case Presentation

An 11‐year‐old mixed‐breed female dog was presented with a subcutaneous edema of the cervical region, resulting in coughing and choking. The physical examination revealed muffled lung sounds in the left pulmonary field and tachypnea. There was no lymph node enlargement or abdominal pain. The chest radiograph demonstrated an extensive mass in the right central/cranial hemithorax, partially extending to the left, displacing mediastinal structures. Additionally, there was luminal narrowing of the trachea and hepatomegaly. The computed tomography revealed an intrathoracic mass measuring 8.9 cm in diameter, likely located in the cranial mediastinum, exhibiting foci of mineralization with evidence of vascular invasion in the cranial vena cava, suggesting cranial vena cava syndrome with edema of the head and neck. There was also extensive subcutaneous edema in the cervical region, displacement of the esophagus, brachiocephalic trunk, left subclavian artery, heart, and aortic arch to the left; additionally, there was incarceration, compression, and leftward displacement of the thoracic trachea. An ultrasound‐guided fine‐needle aspiration (FNA) of the intrathoracic mass was submitted to cytology evaluation (Figure 1).

**FIGURE 1:**
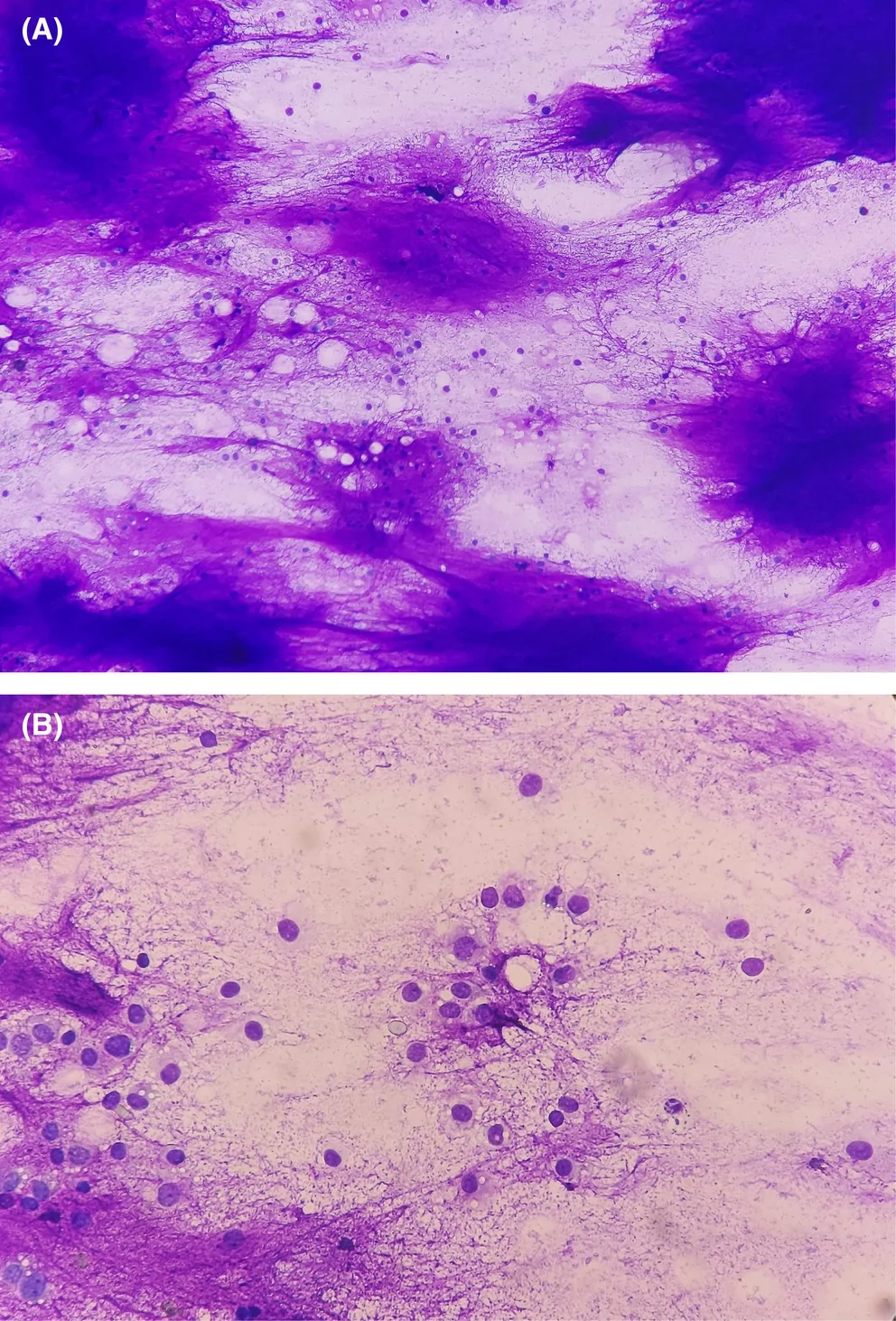
Photomicrograph of a fine‐needle aspirate from an intrathoracic mass in a dog. Occasional cells with indistinct cytoplasm and oval nuclei interspersed with a large amount of eosinophilic amorphous and fibrillar material suggestive of mucinous extracellular. Aqueous Romanowsky stain, (A) ×10 objective, (B) ×40 objective.

### 1.1 Cytologic Interpretation: Mucin‐Producing Mesenchymal Tumor

The cytologic examination revealed scant, individualized, round to spindle‐shaped cells. These cells had moderate, occasionally indistinct, slightly basophilic, and frequently vacuolated cytoplasm. The nuclei were round to oval, predominantly central to but slightly eccentric, with loose chromatin and a single prominent nucleolus. A large amount of homogeneous and fibrillar eosinophilic matrix was noted between the cells. Rare small well‐differentiated lymphocytes and rare clusters of well‐differentiated adipocytes were observed. Cytological smears were stained with Alcian Blue (Figure 2), confirming the mucinous origin of the matrix. Differential diagnoses included myxoma, myxosarcoma, and chondrosarcoma.

**FIGURE 2:**
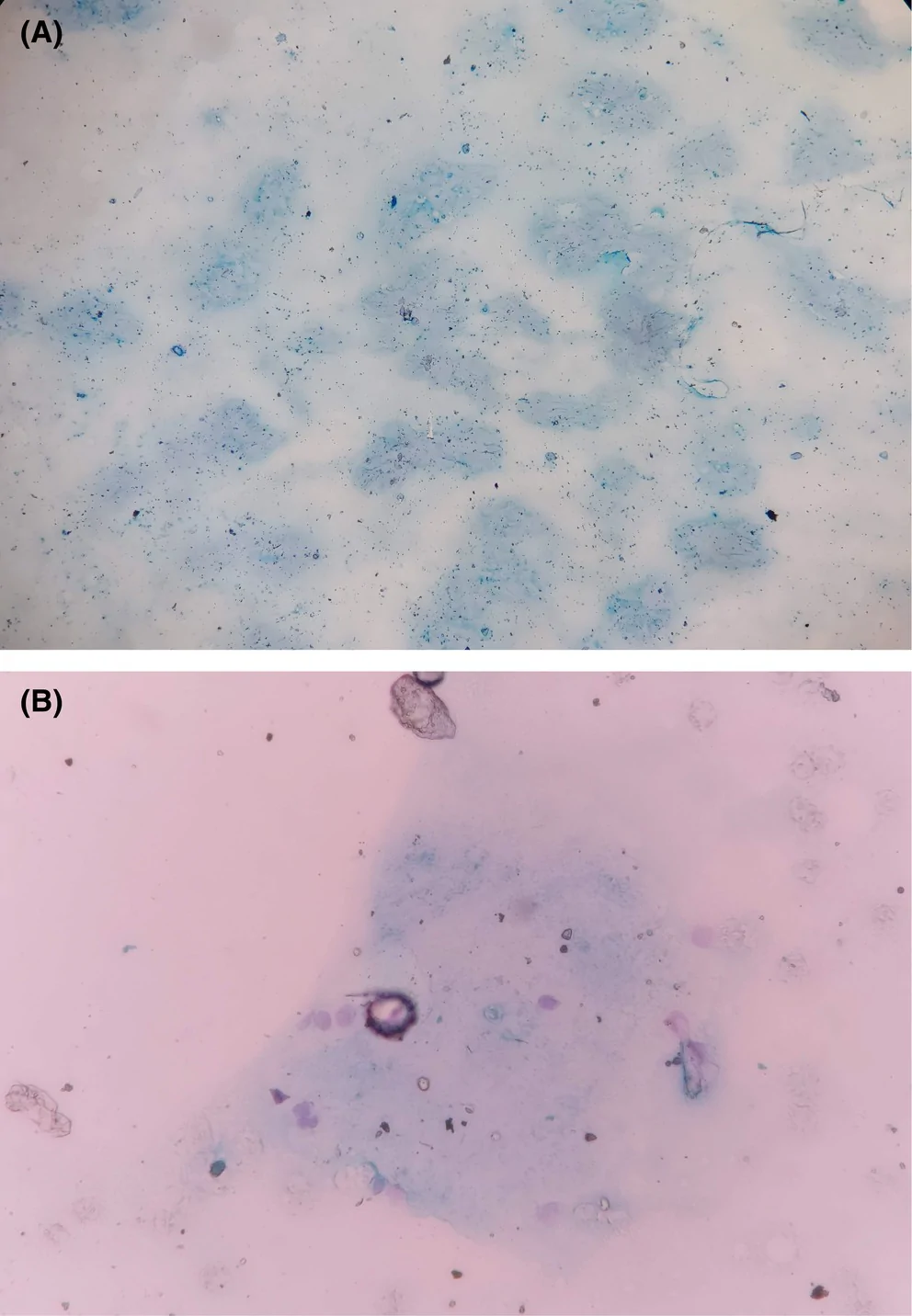
Photomicrograph of a fine‐needle aspirate from an intrathoracic mass in a dog. Mucin deposit staining pale blue, Alcian Blue with hematoxylin, pH 2.0. (A) ×10 objective, (B) ×40 objective.

## 2 Additional Results

Due to the location and extent of the mass, surgical intervention was considered unfeasible, and palliative care was elected. The dog died 1 month after the cytology was collected. A necropsy was conducted, and histopathologic analysis was performed on the mass, esophagus, lung, cranial vena cava, liver, kidneys, lymph node, and heart.

Histopathologic analysis of the mass revealed an atypical proliferation of moderately pleomorphic chondrocytes arranged in a multinodular pattern. The mass was well delineated, non‐encapsulated, and occasionally formed nests, supported by dense fibrovascular stroma. The nuclei were pleomorphic, oval to stellate, small to medium‐sized, hyperchromatic, and occasionally eosinophilic, indicating necrosis. Immature cartilage and myxoid/mucinous matrix, as well as osseous metaplasia, were present. Additionally, there were foci of necrosis and hemorrhage, along with isolated fragments composed of well‐differentiated chondrocytes (Figure 3). A similar neoplastic cellular proliferation was observed in the esophagus and lung. Consequently, the lesion was diagnosed as extraskeletal myxoid chondrosarcoma, likely originating from the trachea, with multiple metastatic foci.

**FIGURE 3:**
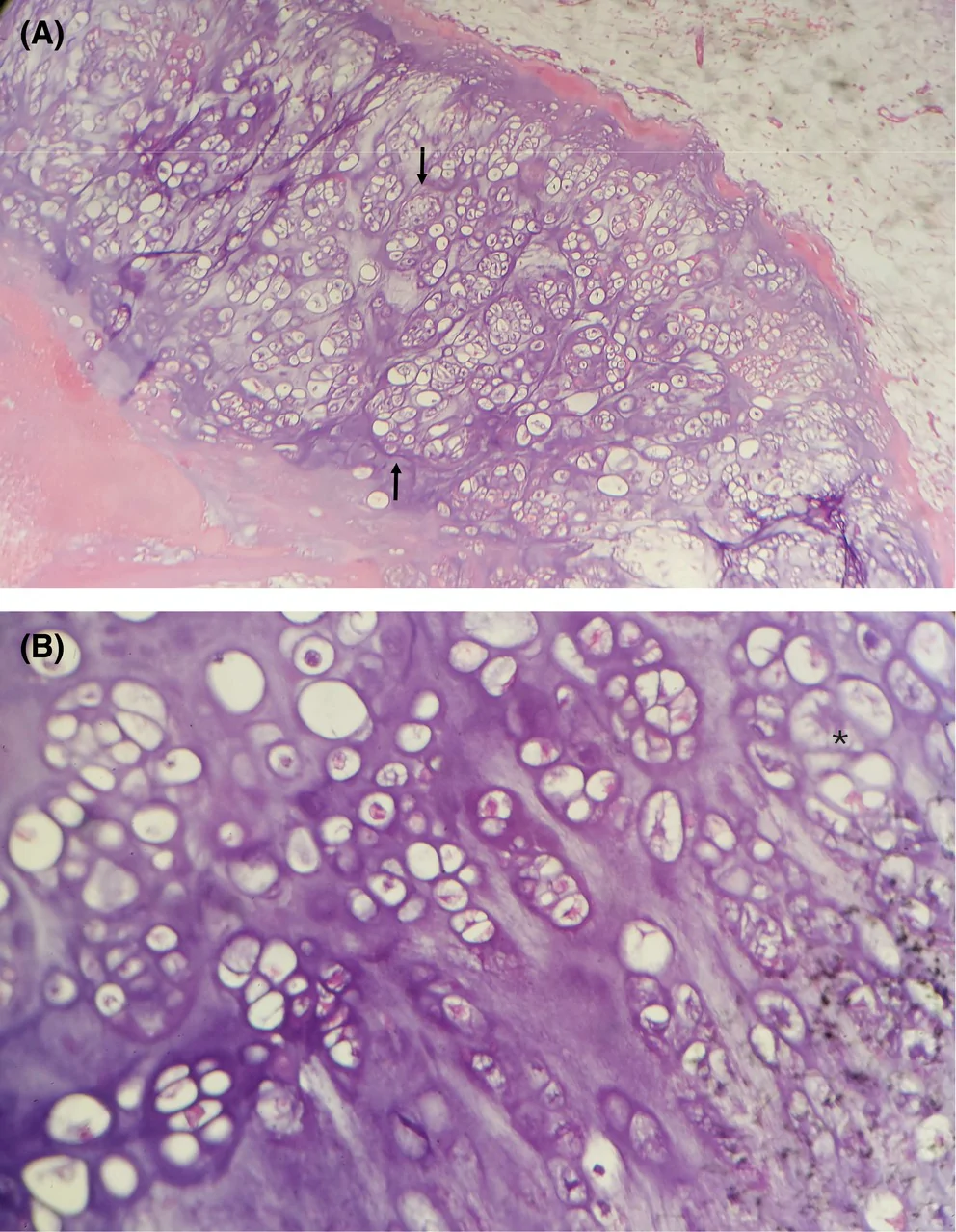
Photomicrograph of a histopathologic section from an intrathoracic mass in a dog with atypical proliferation of moderately pleomorphic chondrocytes, H&E stain, (A) A well‐delineated and non‐encapsulated mass, formed by chondrocyte nests, ×10 objective, (B) Well‐differentiated chondrocytes with nuclei pleomorphic, oval to stellate, small to medium‐sized, and hyperchromatic, ×40 objective.

Extraskeletal chondrosarcoma is a rare tumor in domestic animals, with few reports of the mesenchymal variant and even fewer of the myxoid variant [1]. The origins of extraskeletal chondrosarcomas remain unknown, though it is hypothesized that this tumor may arise from populations of multipotent mesenchymal cells capable of differentiating into cartilage [2].

In line with the cases described in dogs [1] and cats [2], as well as with the human [3] literature, the histologic analysis in this case report reveals the formation of cords and cell nests inserted in a myxoid matrix, cells with moderate, deeply eosinophilic to vacuolated cytoplasm and delicate cytoplasmic extensions, rounded to oval nuclei with uniformly distributed chromatin and small and discrete nucleoli, in addition to low mitotic activity [4]. These mesenchymal cells are dark, uniform in size, and show variable shapes. The cytoplasm is often vacuolated, foamy, and indistinct. Nucleoli are generally not evident [3]. These findings characterize the tumor phenotype recognized in both species.

The challenge in the cytologic diagnosis of this case arose from the undifferentiated cell morphology and the abundant homogeneous fibrillar matrix with features suggestive of a myxoid matrix, which influenced the differential diagnoses. Moreover, the lack of similar clinical and cytologic reports in the literature hindered a more specific diagnosis.

Alcian Blue cytochemical stain was complementary in the diagnosis of mucin‐producing tumors by staining mucins and proteoglycans, and in chondroid tumors by staining acidic polysaccharides, such as glycosaminoglycans and certain types of mucopolysaccharides. However, in human literature, more than 60 neoplastic and reactive entities are classified as predominantly myxoid or containing myxoid areas [5].

In this case, the cytochemical analysis was useful for diagnosis in place of histopathology. Additional immunocytochemical staining with vimentin and S100B (S100 protein) can improve diagnostic specificity by confirming co‐expression of mesenchymal markers in matrix‐producing neoplasms.

This case illustrates that extraskeletal myxoid chondrosarcoma should be considered in the differential diagnosis of mucin‐producing tumors. The definitive diagnosis of extraskeletal myxoid chondrosarcoma in veterinary medicine is based primarily on cytogenetic‐molecular methods. Fluorescent in situ hybridization (FISH), using break‐apart or dual‐fusion probes targeting the NR4A3 gene and its most common partner, EWSR1, allows rapid and sensitive demonstration of the pathognomonic translocation t(9;22)(q22;q12). In addition, RT‐PCR enables the detection and sequencing of fusion transcripts such as EWSR1‐NR4A3, TAF15‐NR4A3, and FUS‐NR4A3, confirming the presence of the rearrangement at the RNA level [1, 2]. In addition to the differential diagnoses considered in this case, it is important to consider other mesenchymal neoplasms with a myxoid component and Alcian blue‐positive matrix. Myxoid liposarcoma, for example, often has an abundant mucopolysaccharide matrix and lipoblasts. Low‐grade myxoid fibrosarcoma has alternating areas of fibrous and myxoid matrix. Myxoid leiomyosarcoma, although rare, shows positivity for smooth muscle actin and desmin in myxoid portions, while myxoid peripheral neural sheath tumor shows S100 positivity in its cellular components. Furthermore, myxoid variants of synovial sarcoma and even myxoid forms of mesothelioma can mimic this histochemical pattern. Additional tests, such as histopathologic analysis and cytogenetic assessments, are essential for the definitive diagnosis of these tumors [1, 3]. The limited literature regarding the cytologic presentation of this tumor underscores the significance of this report.

## Conflicts of Interest

The authors declare no conflicts of interest.
